# A Case of Septum Pellucidum Agenesis in a Patient with Psychotic Symptoms

**DOI:** 10.1155/2020/8935986

**Published:** 2020-01-07

**Authors:** Alexander Kilpatrick, Heela Azizi, Joshua Jay, Cecilia Canale, Jeffrey Balkenbush, Filipa Cardoso, Hashem Kalbouneh, Tasmia Khan, Isaac Kim, Alexa Kahn, Paul Saad, Deepa Nuthalapati, Saravjit Bhatti, Mayra Mejia, Ayodeji Jolayemi

**Affiliations:** ^1^American University of Antigua College of Medicine, Department of Psychiatry, Interfaith Medical Center, Brooklyn, New York, USA; ^2^St. Matthew's University School of Medicine, Department of Psychiatry, Interfaith Medical Center, Brooklyn, New York, USA; ^3^Department of Psychiatry, Interfaith Medical Center, Brooklyn, New York, USA; ^4^Saba University School of Medicine Department of Psychiatry, Interfaith Medical Center, Brooklyn, New York, USA; ^5^Medical University of the Americas, Department of Psychiatry, Interfaith Medical Center, Brooklyn, New York, USA

## Abstract

Agenesis of the septum pellucidum is a rare congenital defect that has been associated with psychiatric disorders, cognitive deficits, learning disabilities, seizures, and neuropsychiatric disturbances. We present the case of a patient with partial agenesis of the septum pellucidum who exhibits disorganized behavior and paranoid and persecutory delusions. We add to the literature of incidental neuropsychiatric symptoms in patients with partial agenesis of the septum pellucidum which is an area that requires further exploration and study. We discuss the implications of these findings in light of previous literature findings.

## 1. Introduction

The implications of agenesis of the septum pellucidum are varied, and its role in neuropsychiatric manifestations is an area to be explored. The septum pellucidum develops embryologically from the primitive lamina terminalis, forming a neural structure located above the fornix and below the body, genu, and rostrum of the corpus callosum, consisting of a thin translucent plate of 2 laminae. “The 1.3-3.0 mm wall [of the septum pellucidum], contains glial cells, some scattered neurons, fiber bundles, and veins that connect with the choroid plexus veins” [1]. The septum pellucidum has connections with the hippocampus via precommissural fornix fibers and the hypothalamus via the medial forebrain bundle of Broca [1]. The absence of the septum pellucidum is a phenomenon that occurs in 2-3 of 100,000 births in the general population [2]. Agenesis may occur as part of trisomy 13, 18, and 21 syndromes as there is a high association between abnormal septum pellucidum and chromosomal abnormalities [3]. Beaton et al. showed that a hemizygotic microdeletion of 22q11.2 leads to septum pellucidum abnormalities, thereby possibly providing a biomarker that is suggestive of atypical brain development that increases the risk for neurobiological disorders [4].

Literature has shown that schizophrenia, affective psychosis, self-mutilation, developmental delays, atypical psychosis, and bipolar disorder have been found in patients that show septum pellucidum agenesis (SPA) on imaging [5–9]. However, no direct correlation can be drawn between neurobiological disorders and SPA, as multiple abnormalities have been seen on imaging in the respective patients. Some of the abnormalities seen on imaging are within the diencephalon region or are midline in general, but specific defects such as corpus callosum dysgenesis, cystic cavum vergae, cavum septum pellucidum (CSP), hippocampal dysgenesis, hydrocephalus, holoprosencephaly, syntelencephaly, septooptic dysplasia, and schizencephaly are also seen [3, 6, 8, 10, 11]. Visualization of all the cytoarchitectural disturbances that exist within the brain may not be possible due to the limitations of MRI [12].

We present the case of a patient who has septum pellucidum agenesis and explore the neuropsychiatric symptomatology and manifestations that she demonstrates.

## 2. Case Presentation

The patient is a 47-year-old Hispanic woman who was brought to the psychiatric emergency department following an episode of disorganized behavior. The patient was unable to provide details regarding the incident that led to her admission. We were able to, however, gain collateral information from the patient's family that she had previously received outpatient psychiatric treatment for severe major depressive disorder with psychotic features, requiring inpatient treatment.

On evaluation, the patient detailed paranoid and persecutory delusions and reported that she felt depressed. She believed that her next-door neighbors were spying on her and attempting to steal her identity in order to humiliate and torture her. Collateral information which was later obtained from her sister, with whom she had been cohabiting, revealed that none of this was true but that she had suffered from depression in the past. The patient self-reported a depressed mood with suicidal ideation, stating that she cried often and had difficulty sleeping. During the intake interview, she exhibited disorganized behavior, paranoid thinking, and tangential thought process, with an irritable affect and no symptoms of mania. The patient denied auditory or visual delusions; however, she had poor insight into her condition. Considering the totality of her symptoms and clinical presentation, she was admitted with a presumptive diagnosis of a depressive disorder with psychotic features.

The patient was started on aripiprazole 5 mg daily targeting psychosis which was uptitrated to 10 mg after day four of treatment. For depression, bupropion 150 mg daily was administered. On day four of admission, trazodone 50 mg was initiated for the insomnia symptoms and for the augmentation of an antidepressant effect. Trazodone was later increased to 100 mg at bedtime. As she had medical comorbidities of neuralgia and restless leg syndrome, she was prescribed gabapentin at a starting dose of 300 mg three times daily with an as needed basis prescription of clonazepam 2 mg PO once nightly should the symptoms of neuralgia and restless leg syndrome become unbearable.

Within the first week of admission, the patient's symptoms of depression began to improve. She also endorsed improvement in mood symptoms and insomnia. Additionally, the patient had exhibited psychomotor retardation, poor concentration, and low energy, all of which were noted to be gradually improving by the end of her first week of admission. She was mostly compliant with her medications but occasionally refused medications believing they were causing her to hear voices. After 37 days, we observed the patient gained insight that the people around her were not actually talking but that she was experiencing auditory hallucinations. During hospital course, she had improved awareness into her condition compared to her state at admission where she denied auditory hallucinations and may have not been able to differentiate voices she heard in her head from those she heard in reality. Despite this, she continued to endorse paranoid and persecutory delusions throughout her inpatient treatment, stating other patients were conspiring against her, nursing staff were not interested in helping her, and that her family members were at risk of being assassinated when they came to visit her. Due to the patient being compliant with her medications only some of the time, but showing improvements when she was compliant, it was decided that the current medication schedule would not change. This would serve as a bridge to eventually switch the patient to a long-term injectable, where compliance only became an issue on a monthly not daily basis.

**Figure 1:**
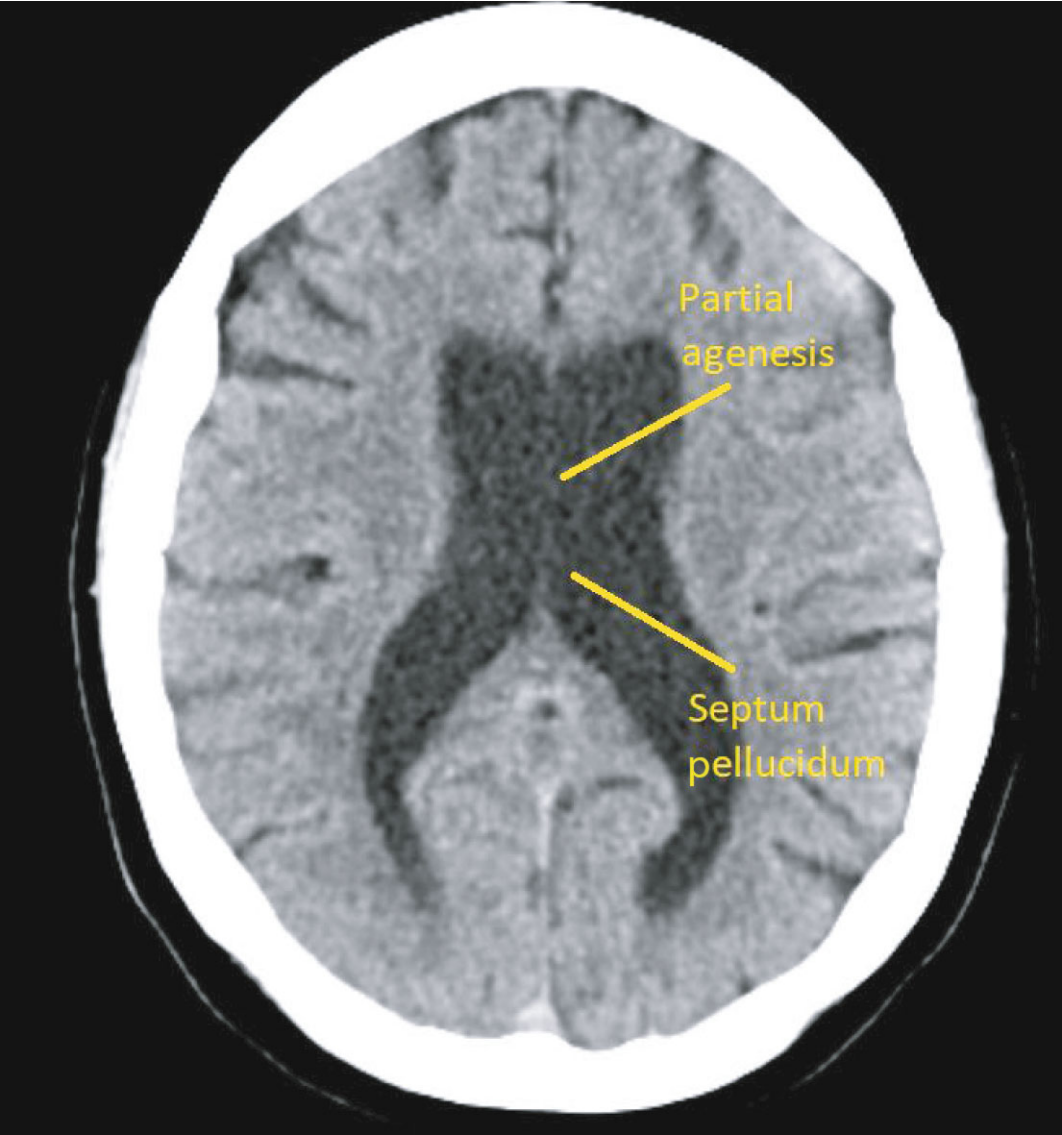
Axial MRI showing septum pellucidum agenesis.

**Figure 2:**
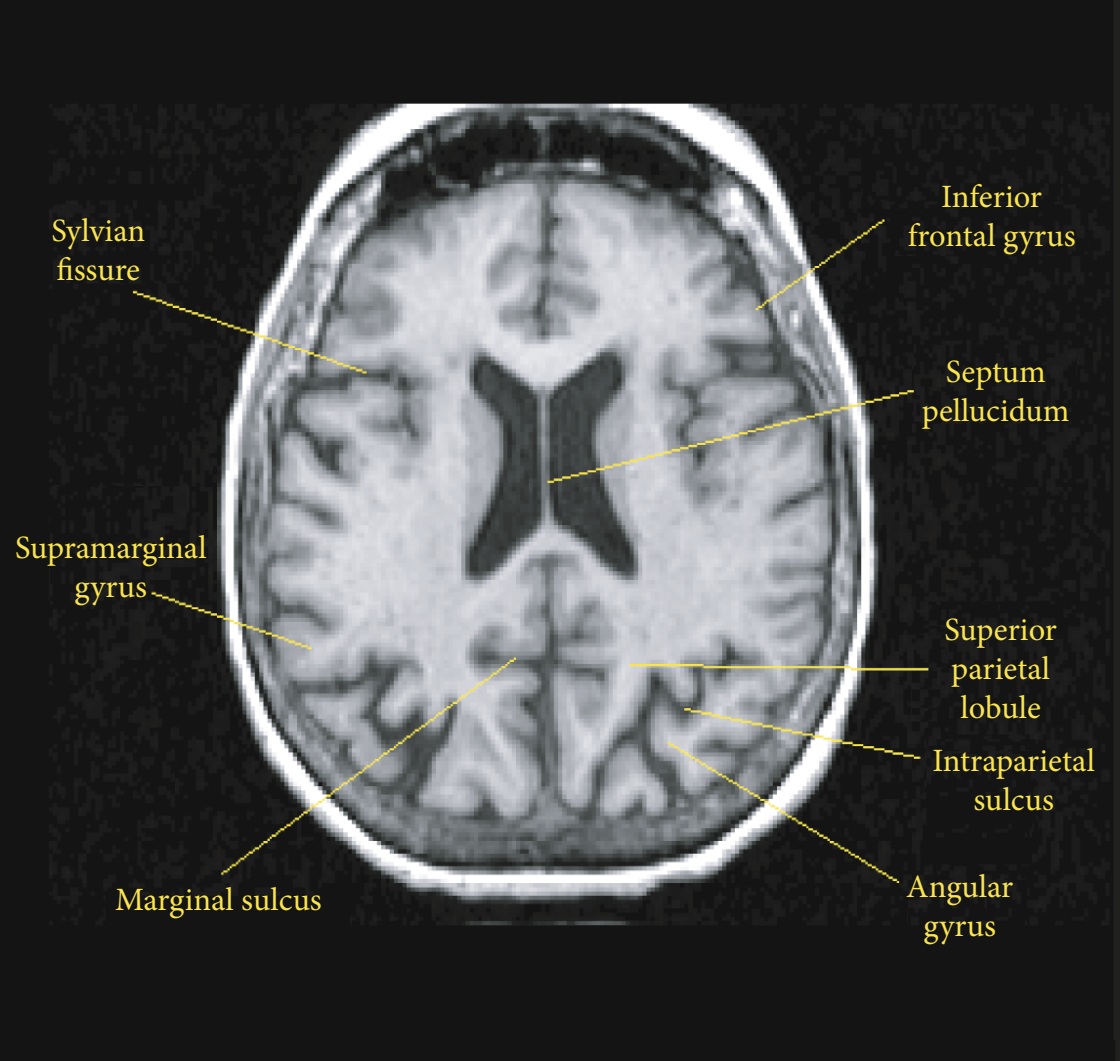
Normal axial MRI.

Due to the patient's persistent psychotic symptoms, a head CT was performed and revealed chronic involutional and white matter changes and no acute intracranial process. Therefore, a follow-up MRI was performed.  Figure 1 shows partial agenesis of the septum pellucidum and a thin, normal variant corpus callosum, ventriculomegaly, along with mild cortical atrophy as compared to the normal, seen in Figure 2.

At day 40 of admission, the patient's depressive symptoms had resolved, but her paranoid delusions persisted. Taking her clinical presentation at that time and her previous psychiatric history into account, the diagnosis of Unspecified Schizophrenia Spectrum and Other Psychotic Disorder was determined to be the most likely explanation. However, considering the patient's MRI results, this diagnosis may be due to the partial agenesis of her septum pellucidum. She was judged to be stable enough for outpatient treatment, and her family members confirmed that she had reached her baseline, which still included paranoia. The patient was discharged with a referral to the hospital's outpatient clinic but, at that time, did not have insurance to follow up with outpatient treatment; therefore, she was referred to another treatment program. One month after being discharged from the hospital, the patient was contacted to see the status of her insurance. At that point in time, the patient was still attending the same outpatient treatment program.

## 3. Discussion

The patient presented in this study was found to be missing part of her septum pellucidum. The patient presented with mood and psychotic symptoms long before structural abnormalities within the brain were found on CT as an incidental finding. However, these abnormalities may have been present at an earlier time. This incidental finding leads to questions about a possible relationship between this brain structure and functions in neuropsychiatric pathology. It is important to point out that other structural abnormalities of septum pellucidum do exist and have been linked to neuropsychiatric disorders—particularly schizophrenia. For one, CSP is an embryological disorder of the septum pellucidum where an enlarged slit-like space occurs between the two leaflets [13]. Cystic cavum vergae is another abnormality that is associated with the septum pellucidum. It is a posterior extension of the septum pellucidum and is the persistence of an embryological fluid-filled space between the septum pellucidum leaflets [10]. Additional related abnormalities that have been cited in the literature are syntelencephaly, septooptic dysplasia, hippocampal dysgenesis, schizencephaly, holoprosencephaly, noncleavage of the thalamus, noncleavage of the hemispheres, septooptic dysplasia, and hydrocephalus [3, 6, 8, 11, 14].

There are studies that show an association between septum pellucidum abnormalities (CSP and agenesis) and neuropsychiatric disorders. Literature that does exist mentions schizophrenia, affective psychosis, self-mutilation, developmental delays, atypical psychosis, and bipolar disorder as possible manifestations of brain pathologies. These diagnoses match with the interrelated functions of the septum pellucidum which are consciousness, sleep, emotional response to the environment, mental processes of self-maintenance, food-finding, sexuality, autonomic-vegetative adaptation modes for homeostasis, fight and flight, and species maintenance [1]. For instance, 77 patients with schizophrenia were studied and it was found that the larger the CSP, the higher the reported clinical symptom ratings [15]. In another study, 62 patients suffering from schizophrenia were compared to a control group and were found to have a higher prevalence of CSP [16]. A further study pointed out that a high prevalence of CSP has been reported in first episode psychoses with mood symptoms, bipolar mood disorders, and schizotypal personality disorders [17]. However, the studies reported also indicated other brain abnormalities such as agenesis of the corpus callosum. It is unclear in those cases the contributory role the other abnormalities played in the patients' presentations. Future studies may be needed to compare patients with isolated septum pellucidum abnormalities with patients who have additional abnormalities in other brain areas.

Management and response of patients with structural brain abnormalities and associated neuropsychiatric conditions are lacking in the available literature. Of the few articles that mention any treatment, there is a fairly good psychiatric response rate with decreased symptoms when patients are treated with typical antipsychotics [6, 18].

Further research is encouraged to compare treatment outcomes in schizophrenic patients with structural brain abnormalities and patients without. CSP may play a crucial role in early neurodevelopmental wiring, predisposing the patient to neuropsychiatric behavior changes in the future. It is not clear whether septal pellucidum lesions or defects directly cause neuropsychiatric disorders or are merely coexisting pathologies.

## 4. Conclusion

Our case report indicates the symptoms of mood disturbances and psychosis as seen in the criteria for a diagnosis of Unspecified Schizophrenia Spectrum and Other Psychotic Disorder [19]. Similar symptoms in patients with incidental findings of isolated septum pellucidum abnormalities can present similarly to patients with septum pellucidum abnormalities and additional brain imaging findings such as agenesis of the corpus callosum. Future studies may be needed to compare symptoms of patients with septum pellucidum abnormalities isolated and patients with septum pellucidum abnormalities and other findings.
